# *APOE3* Christchurch modulates β-catenin/Wnt signaling in iPS cell-derived cerebral organoids from Alzheimer’s cases

**DOI:** 10.3389/fnmol.2024.1373568

**Published:** 2024-03-20

**Authors:** Paula Perez-Corredor, Timothy E. Vanderleest, Guido N. Vacano, Justin S. Sanchez, Nelson D. Villalba-Moreno, Claudia Marino, Susanne Krasemann, Miguel A. Mendivil-Perez, David Aguillón, Marlene Jiménez-Del-Río, Ana Baena, Diego Sepulveda-Falla, Francisco Lopera, Yakeel T. Quiroz, Joseph F. Arboleda-Velasquez, Randall C. Mazzarino

**Affiliations:** ^1^Schepens Eye Research Institute of Mass Eye and Ear and Department of Ophthalmology at Harvard Medical School, Boston, MA, United States; ^2^Vacano Informatics LLC, Arvada, CO, United States; ^3^Massachusetts General Hospital and Department of Neurology at Harvard Medical School, Boston, MA, United States; ^4^Molecular Neuropathology of Alzheimer’s Disease, Institute of Neuropathology, University Medical Center Hamburg-Eppendorf, Hamburg, Germany; ^5^Institute of Neuropathology, University Medical Center Hamburg-Eppendorf, Hamburg, Germany; ^6^The Neuroscience Group of Antioquia, University of Antioquia, Medellín, Colombia; ^7^Massachusetts General Hospital and Department of Psychiatry at Harvard Medical School, Boston, MA, United States

**Keywords:** Wnt signaling, ApoE, iPS cells, CRISPR, Alzheimer’s disease, ApoE Christchurch, Presenilin

## Abstract

A patient with the *PSEN1* E280A mutation and homozygous for *APOE3* Christchurch (*APOE3Ch*) displayed extreme resistance to Alzheimer’s disease (AD) cognitive decline and tauopathy, despite having a high amyloid burden. To further investigate the differences in biological processes attributed to *APOE3Ch*, we generated induced pluripotent stem (iPS) cell-derived cerebral organoids from this resistant case and a non-protected control, using CRISPR/Cas9 gene editing to modulate *APOE3Ch* expression. In the *APOE3Ch* cerebral organoids, we observed a protective pattern from early tau phosphorylation. ScRNA sequencing revealed regulation of Cadherin and Wnt signaling pathways by *APOE3Ch*, with immunostaining indicating elevated β-catenin protein levels. Further *in vitro* reporter assays unexpectedly demonstrated that ApoE3Ch functions as a Wnt3a signaling enhancer. This work uncovered a neomorphic molecular mechanism of protection of ApoE3 Christchurch, which may serve as the foundation for the future development of protected case-inspired therapeutics targeting AD and tauopathies.

## Introduction

Alzheimer’s disease (AD) is the most common cause of dementia among older adults. AD affects an estimated 55 million people worldwide with numbers expected to exceed 152 million people by the year 2050 (Patterson, 2018). AD is characterized by the formation of amyloid plaques and tau tangles in the brain as well as calcium and mitochondrial dysregulation that manifests in neuronal death and memory deficits (Nunomura et al., 2001; Mosconi, 2013; Knopman et al., 2021). Autosomal dominant Alzheimer’s disease (ADAD) accounts for approximately 1% of diagnosed patients (Sims et al., 2020), with approximately 70% of ADAD patients having a Presenilin-1 (*PSEN1*) mutation (Sun et al., 2017).

Recently, a member of the Colombian *PSEN1* E280A (Paisa) kindred was identified as being resistant to ADAD. Carriers of the *PSEN1* E280A mutation develop mild cognitive impairment at 43–45 and dementia at 49–50 years of age (95% confidence intervals); the identified female patient did not develop mild cognitive impairment until her seventies. She had very limited levels of tau pathology, neuroinflammation, and neurodegeneration but extremely high levels of amyloid plaque burden (Arboleda-Velasquez et al., 2019; Sepulveda-Falla et al., 2022; Lopera et al., 2023).

She was also found to be homozygous for the Apolipoprotein E3 Christchurch (*APOE3Ch*) variant (R136S), which was identified as a candidate variant responsible for her resistance to ADAD (Arboleda-Velasquez et al., 2019). Genetic imputation of causality could not be confirmed because only a single *APOE3Ch* homozygote case with resistance was identified. We hypothesized that the iPS system devised here would be an informative model to identify biological pathways influenced by the Christchurch variant in *APOE* that will shed light on her remarkable AD resistance. In this study, we used cells from the resistant and a non-resistant patient to generate iPS cells, used genomic editing to introduce or remove the *APOE3Ch* or *PSEN1* E280A mutations, and identified the Cadherin/Wnt/β-catenin signaling pathways as plausibly regulated by the *APOE3Ch* variant.

## Methods

### Patient selection and sample collection

We have selected two patients for this study, and they will be known in this study as Patient α and Patient ω to ensure patient privacy. Patient α was previously described as being a protected patient from familial Alzheimer’s disease (Arboleda-Velasquez et al., 2019). Patient α was part of the Paisa *PSEN1* E280A kindred in her seventies at a time of mild cognitive impairment. She was found to have the *APOE3* R136S Christchurch variant that provided her resistance to Alzheimer’s development. Patient ω was also selected as a Paisa kindred female with the development of ADAD at the expected age of onset and which PET imaging data were available with expected brain pathology.

Blood samples from each individual were obtained by venipuncture. Peripheral blood mononuclear cells were separated by Ficoll–Hypaque 1077 and submitted for reprogramming and genetic editing.

### *In vivo* neuroimaging

Structural magnetic resonance imaging (MRI), Pittsburgh compound B (PiB), and Flortaucipir (FTP) positron emission tomography (PET) were performed at Massachusetts General Hospital, as described elsewhere (Quiroz et al., 2018). In brief, MRI images were processed with FreeSurfer (FS, v 6.0) to identify surface boundaries and standard regions of interest (Desikan et al., 2009). PET data were acquired and processed according to previously published protocols (Johnson et al., 2016), whereas PiB data were expressed as distribution volume ratios (DVR, Logan, 0–60 min) and FTP as standardized uptake value ratios (SUVR, 80–100 min), both using cerebellar gray matter as the reference region. PET images were affine co-registered to each subject’s T1 images and visualized using FS surface projections (sampled at the midpoint of gray matter, surface-smoothed 8 mm). No partial volume correction was applied to PET images for the purposes of this study.

### Reprogramming and genetic editing

Cell services were performed using the Harvard Stem Cell Core: whole blood was reprogrammed via Cytotune 2.0 (Thermo Fisher), and colonies were allowed to grow and were then assessed for the iPS markers SSEA, Oct4, Tra-1-60, and Nanog using immunocytochemistry and qPCR for trilineage. Successful screened colonies were then processed further for genetic editing. Guide RNA (gRNA) and single-stranded oligodeoxynucleotide (ssODN) sequences were determined (Supplementary Table 1) using the CRISPOR suite through the highest specificity score and lowest off-target homology, and then, colonies were karyotyped. Normal karyotype colonies were sequenced for their inclusion of the desired genetic mutation, and isogenic controls were also selected (Supplementary Figure 2).

### Cell culture

Reprogrammed and edited cells were cultured on human embryonic stem cell (hESC)-qualified Matrigel (Corning #354277) using mTeSR Plus (StemCell Technologies #100-0276) supplemented with the antimicrobial Normocin (Invivogen #ant-nr-1) and clump passaged weekly using ReLeSR (StemCell Technologies #05872) according to the manufacturer’s recommended protocols. Cell clumps were sparsely plated to allow for easy physical removal of spontaneously differentiated cells. Cells were grown at 37°C and 5% CO_2_.

### Differentiation

Cells were cultured as described above. Regions of spontaneously differentiated cells were identified and physically removed. Organoids were made using Stemdiff Cerebral Organoid Kit (StemCell Technologies #08570) and Normocin using the manufacturer’s recommended protocol; however, iPS cell spheroids were made using EB formation media (Stem Cell Technologies #05893) with Normocin. In brief, cells were detached to single cell using Accutase (StemCell Technologies #07920) and counted (Countess II) using Trypan Blue. In total, 9,000 cells/well were plated into low retention 96-well U-bottom plates (S-Bio #MS-9096UZ) placed in EB formation media with γ-27632 (ATCC #ACS-3030) in addition to regular fresh media. After 5 days, spheroids were transferred to a 24-well flat bottom low retention plate (Corning #3473) to induce differentiation. Organoids were then embedded into individual hESC-qualified Matrigel droplets and plated into 6-well flat bottom low retention plates (Corning #3471) for expansion. Organoids were then placed onto a shaker plate inside the incubator with regular media exchanges (2–3 days) until downstream analysis 29 days after initial EB spheroid formation.

### Immunostaining

Organoids for immunofluorescence analyses were fixed in 4% PFA for 2 h, then washed in dPBS thrice, and then infiltrated with 30% sucrose until they dropped then frozen in OCT and sectioned at 15 μm. Sections were washed with 1xdPBS and blocked for 1 h (PBS, 3% BSA, 0.2% Triton X-100, and 0.02% sodium azide). Middle sections were preferentially selected for IF analysis. Then, they were incubated in primary antibody diluted in blocking buffer using the following primary antibodies: anti-Phospho-Tau (Ser396) (1:500, 44-752G, Invitrogen), anti-β-catenin (E-5) (1:50, sc-7963, Santa Cruz), and anti-Reelin (CR-50) (1:100, D223-3, MBL Life Science) overnight at 4°C. After washing the sections with 1xdPBS, they were incubated in the following secondary antibodies: Donkey anti-rabbit IgG Alexa Fluor 647 (1:500, A-31573, Thermo Fisher) and Donkey anti-mouse IgG Alexa Fluor 488 (1:500, A-21202, Thermo Fisher). DAPI solution was used for nuclei staining. Images were taken at 63X magnification using a ZEISS Axioscope digital microscope. A total of 3–4 organoids were imaged and used for quantification. The Shapiro–Wilk normality test was performed followed by a one-way ANOVA and Tukey’s multiple comparison for *post-hoc* analysis.

Immunofluorescence staining was performed on the formalin-fixed paraffin-embedded (FFPE) brain tissue from the frontal cortex, hippocampus, and occipital cortex from a previously described *PSEN1* E280A carrier homozygous for the *APOE3Ch* mutation (Sepulveda-Falla et al., 2022). In total, 4-μm thick sections were mounted on Superfrost plus slides and further processed for immunofluorescence staining for β-catenin (1:200; 05-665, Sigma Aldrich) and RNA binding protein, fox-1 homolog 3 (NeuN, 1:200; 26975-1-AP, Protein Tech). After deparaffinization, heat-induced epitope retrieval was performed using R-Universal buffer (AP0530-500; Aptum Biologics, Southampton, United Kingdom) in a pressure cooker for 20 min, and sections were then blocked for 1 h with blocking medium (MAXblock™, 15252; Active Motif GmbH) followed by incubation with primary antibodies at 4°C overnight. For the detection of specific binding, secondary antibodies were incubated at room temperature for 1 h. After washing, mounting was performed with 4′,6-Diamidino-2-phenylindole (DAPI) Fluoromount-G for nuclear counterstaining. High-resolution images were obtained with a Leica TCS SP8 confocal laser scanning microscope (Leica Microsystems, Mannheim, Germany) using a 20X immersion oil lens objective.

### scRNA-seq

Six organoids from each individual cell line (eight lines in total) were pooled and processed using MACS papain neural tissue dissociation kit (Miltenyi Biotec #130-092-628) according to the manufacturer’s protocol and resuspended in 0.22 μm filter-sterilized dPBS 0.04%BSA solution. Cells were serially filtered through a 70-μm (Miltenyi Biotec #130-110-916) and then a 40-μm (Bel-Art #H136800040) strainer to remove clumped cells. Samples were kept on wet ice and assessed at the BioMicroCenter core facility (Massachusetts Institute of Technology) for viability, cell density, and quality. Samples were processed on 10X Genomics Chromium Controller at the BMC Core facility. Sequences were then processed through the 10X Genomics Cell Ranger Suite.

### Transcriptomic analysis

The h5 files were read with the Seurat Read10X_h5() function [Seurat (Hao et al., 2021), an R package for scRNA-seq clustering and integration]. The DoubletFinder (McGinnis et al., 2019) doubletFinder_v3() function was employed to identify and remove likely multiplets in each sample, and the predicted multiplet rate was 0.8% per “Targeted Cell Recovery” of 1,000 cells (Chromium). QC cutoffs were employed to remove cells with nFeature_RNA > 8,000 or < 200, nCount_RNA > 50,000, or percent.mito >20%. Sample subsets of 6,400 cells were processed via DietSeurat() (default values) and saved as RDS files.

Sample integration was accomplished by merging Seurat objects with merge(), v.1 SCTransform() (Hafemeister and Satija, 2019) with FindClusters (resolution = 0.3) and Harmony integration [RunHarmony()] (Korsunsky et al., 2019). Seurat objects were saved as RDS files.

Aggregate gene expression [AverageExpression()] in clusters was employed to compare gene expression between samples (log2 adjusted sample ratios).

Comparison groups were established (Table 1) reflecting log2(fc > 1 and < −1) and pathway analysis run using PANTHER Pathways Overrepresentation Test version (PANTHER version 17.0 released 2022-02-22) with *Homo sapiens* (all genes in database) reference genome using Binomial test type and Bonferroni correction.

**Table 1:** Cell lines used for the study.

Reprogrammed cell line	Base genotype	CRISPR target	Mutation success	Final genotype
iPS patient α	E3Ch PS1mut	PS1mut → PS1WT	Successful	E3Ch PS1WT
		PS1mut → PS1WT	Unsuccessful	E3Ch PS1mut
iPS patient α	E3Ch PS1mut	E3Ch → E3WT	Successful	E3WT PS1mut
		E3Ch → E3WT	Unsuccessful	E3Ch PS1mut
iPS patient ω	E3WT PS1mut	PS1mut → PS1WT	Successful	E3WT PS1WT
		PS1mut → PS1WT	Unsuccessful	E3WT PS1mut
iPS patient ω	E3WT PS1mut	E3WT → E3Ch	Successful	E3Ch PS1mut
		E3WT → E3Ch	Unsuccessful	E3WT PS1mut

Patient cells were reprogrammed to generate iPS cells and then subjected to CRISPR gene editing to produce the cell lines used for the study. For this table, E3Ch, APOE3Ch; E3WT, APOE3WT; PS1mut, PSEN1 E280A; and PS1WT, PSEN1WT.

Gene Set Enrichment Analysis (GSEA) (Subramanian et al., 2005) was performed using fgsea to facilitate cell type identification. The MSigDB (v2023.1.Hs) C8 cell type signature gene set database^1^ was queried (after filtering to retain only brain-relevant entries; La Manno et al., 2016; Fan et al., 2018; Zhong et al., 2018; Cao J. et al., 2020). NES (enrichment score normalized to mean enrichment of random samples of the same size) scores ≥7.5 were considered significant and informed cell type assignment. Clusters were further classified by general cell type into “superclusters.” These are “neuronal,” “neuroblast,” “radial glial,” “progenitor,” “oligodendrocyte,” and “neuro endothelial.” We also employed SCSA (Cao Y. et al., 2020; https://github.com/bioinfo-ibms-pumc/SCSA) using output from the Seurat FindAllMarkers function and the most recent SCSA database (whole_v2.db) to validate cell type identifications based on GSEA, and we refined our list to designate clusters 5 and 7 as “radial glia/early astrocyte” based on this analysis. Post-hoc cell identities were determined for transcript expression of TUBB3 for neuronal and HES1, FABP7, and VIM for radial glia.

Monocle (Qiu et al., 2017) was employed for single-cell trajectory analysis and identification of genes that change as a function of pseudotime. The Seurat object from the SCTransform/Harmony analysis (see above) was converted to a Monocle3 cell dataset and processed as described.^2^

### Immunofluorescence image quantification

Rosette β-catenin quantification was implemented in MATLAB 2021a with the use of the Image Processing Toolbox. We quantified the average channel intensity in two types of rosette regions of interest (ROIs): the Body and the Ribbon. The masks (or binary image representations) of the Body and the Ribbon were produced via a combination of hand-drawing (to get the outer boundary and inner luminal boundary lines) and Otsu thresholding to remove background pixels from the hand-drawn regions. The hand-drawn mask for the rosette body was defined as the region between the inner and outer boundaries eroded by 1.8 μm (to ensure that the Ribbon and Body’s outer edges were not included in the Body ROI). The rosette ribbon ROI was defined as the inner luminal boundary line dilated by 1.8 μm to approximately capture the full ribbon thickness.

Rosette area was measured by summing the number of pixels within the rosette Body and Ribbon ROIs. The rosette aspect ratio was defined as the major axis length divided by the minor axis length. We used MATLAB function *regionprops* to measure the major and minor axis lengths.

In both pTau S396 and Reelin fluorescence imaging, background intensity was subtracted via a top-hat filter to remove non-uniform background illumination. Top-hat filtering was accomplished with a large structuring element (54 μm radius disk) so as not to remove the signal from large features of interest. After background removal, we used Otsu’s threshold method to find the foreground and computed the mean intensity over the foreground.

### Wnt signaling reporter assay

TCF/LEF Reporter HEK 293 cell line (BPS Bioscience #60501) was designed to stably contain firefly luciferase under the control of a TCF/LEF reporter element and commercially validated against Wnt3a (RND systems #5036-WN-010). Wnt ligands huWnt16b (RND Systems #7790-WN-025) and hu/msWnt5a (RND Systems #645-WN-010) were also tested. Recombinant ApoE proteins were sourced as a fee-for-service from Innovagen AB produced in *E. coli*. We performed this assay according to the BPS Bioscience recommended protocol. In short, 35,000 cells/well were plated in a 96-well white wall clear bottom TC plate. Cells were treated with a final concentration of 10 μM LiCl in media overnight. All wells were treated with LiCl, including vehicle control. The next day, test compounds were sterile prepared in assay media and incubated at room temperature for 15 min and then added to wells in triplicate to final concentrations indicated. Treated cells were incubated at 37°C and 5%CO_2_ for 5 h. Media were removed, and wells were washed with 1x dPBS. Firefly luciferase was assessed using a commercially available kit (Promega #E1910) and measured on a BioTek Synergy H1 microplate reader. Data were analyzed via GraphPad PRISM and statistical significance using a one-way ANOVA and *post-hoc* Tukey’s test to consider a *p*-value of <0.05 significance.

## Results

### Patient cases selected for analysis

**Figure 1:**
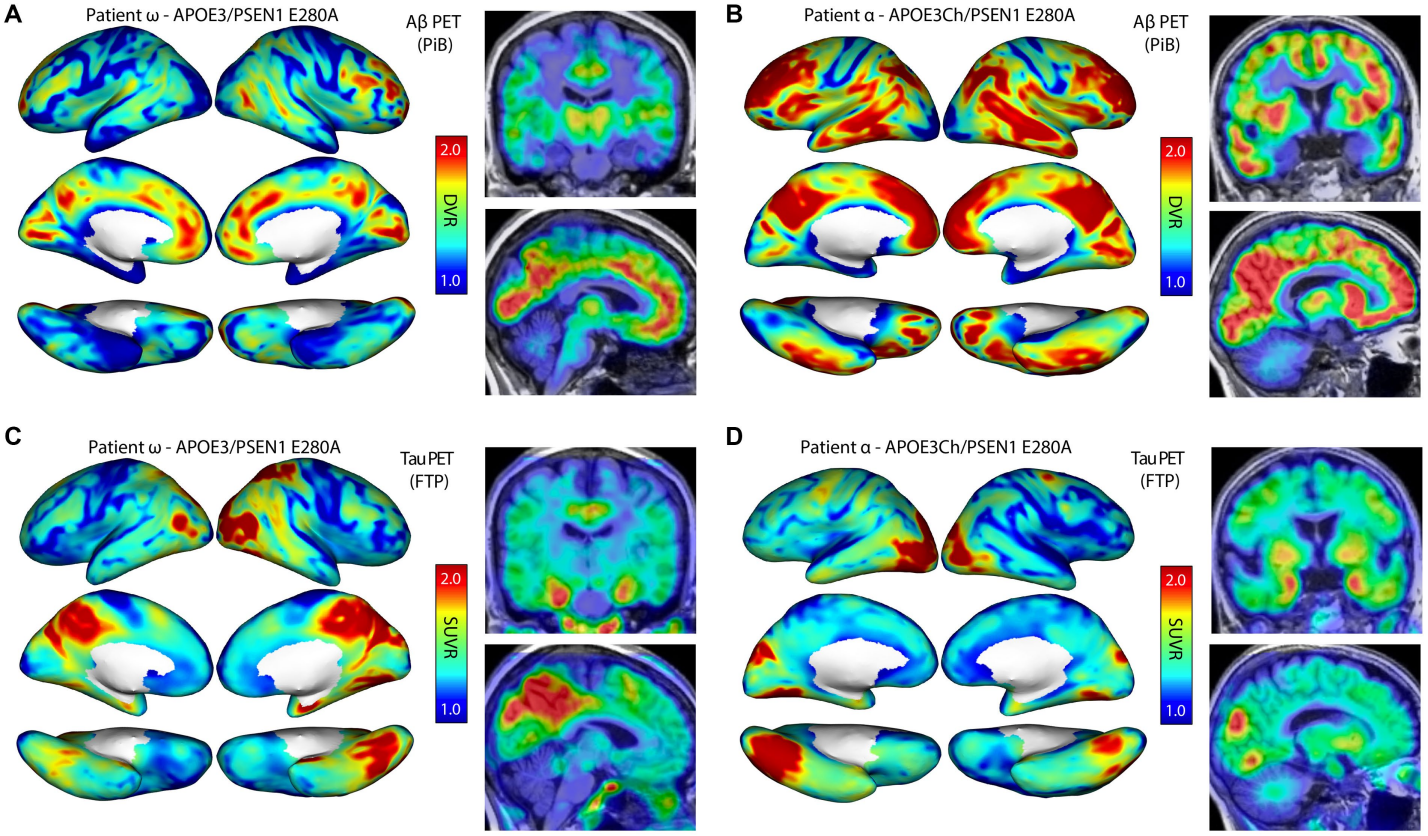
Autosomal dominant Alzheimer’s disease (ADAD) patient *in vivo* neuroimaging. Patient ω is a non-protected, Paisa kindred control patient and was scanned for amyloid β **(A)** and tau burden **(C)**. Patient α is an AD-protected Paisa kindred patient and was previously described and scanned for amyloid β **(B)** and tau burden **(D)** (Arboleda-Velasquez et al., 2019). Scans indicated elevated amyloid β burden in patient α **(B)** compared to patient ω **(A)**. Tau burden was elevated in patient ω **(C)** over patient α **(D)**, with a marked increase in medial temporal and parietal regions. PiB, Pittsburgh Compound B; FTP, Flortaucipir; DVR, Distribution volume ratio; and SUVR, Standardized uptake value ratio.

We identified two informative patients for this study (named α and ω to protect privacy): Patient α was previously described as the homozygote *APOE3Ch* protected case (Arboleda-Velasquez et al., 2019), and Patient ω who was not previously described was selected as a control for this study. Both individuals are from the Paisa *PSEN1* E280A kindred and women, though not closely related. Patient α developed mild cognitive impairment (MCI) in her seventies, suggesting protection against ADAD, while Patient ω developed MCI and dementia in her forties, as expected for the Paisa kindred. PET imaging of each patient was performed (Figures 1A–D) to evaluate amyloid and tau burden. Pittsburgh compound B PET imaging for amyloid plaques revealed elevated burden in Patient α versus Patient ω, while [^18^F]Flortaucipir PET imaging displayed a significant burden in the medial temporal and parietal regions of Patient ω, which is markedly reduced in Patient α. Patient ω was selected as an appropriate control for this experiment based on the following criteria: (1) being a *PSEN1* E280A mutation carrier, (2) being symptomatic, (3) having PET imaging data available for this patient, (4) being female, (5) having a *APOE3/3* wild-type genotype, and (6) having provided informed consent for this study. Taken together, these individuals were selected as an informative pairing for protected and non-protected cases.

### Patient-derived iPS cell generation and gene editing

We generated patient-derived iPS cell lines to examine the effects of the *APOE3Ch* R136S and *PSEN1* E280A mutation (Supplementary Figure 1A). Patient blood samples were successfully reprogrammed and subcloned, as tested by immunofluorescence (IF) staining of pluripotency markers (Supplementary Figure 1B). Reprogrammed subclones were then edited using CRISPR/Cas9 to knock in gene variants to both add and remove the putative *APOE3Ch* protective factor as well as remove the *PSEN1* E280A AD causality factor, generating eight cell lines for the study (Table 1; Supplementary Figure 1A).

Sanger sequencing was performed and successful genetically edited cells were selected while isogenic controls were also selected from reprogrammed cells that underwent the CRISPR editing process but were unsuccessful in genomic editing (Supplementary Figure 2). Altogether, eight cell lines were generated for this study (four from each case). Karyotyping was performed, and all cell lines generated showed 46 chromosomes without overt abnormalities (Supplementary Figure 1C). Cell lines showed success for trilineage; embryoid bodies were formed and differentiated toward ectoderm, mesoderm, and endoderm for 2 weeks and assessed by a three gene qPCR panel: ectoderm—EN1, MAP2, and NR2F2, mesoderm—SNAIL2, RGS4, and HAND2, and endoderm—SST, Klf5, and AFP (Data available on request).

### *APOE3* iPS cell cerebral organoids produce differential phospho-tau patterns

**Figure 2:**
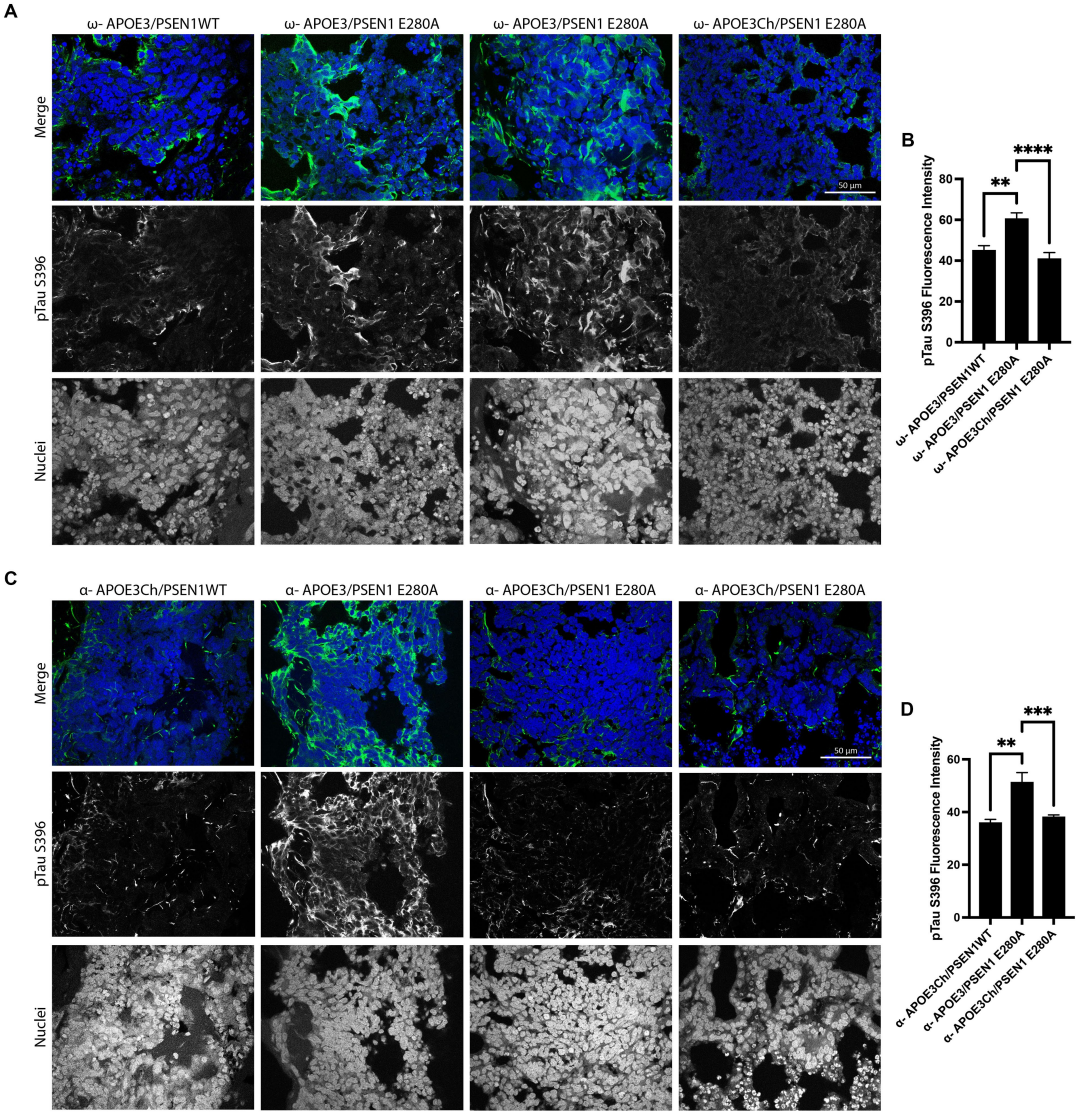
*APOE3Ch* decreases pTau S396. Cerebral organoids were formed and stained for nuclei and pTau S396 and imaged at 63X. Representative images from patient ω. **(A)** and patient α **(C)** depicted, scale bars = 50 μm. pTau S396 signal intensity was measured, and isogenic controls for each individual patient were averaged together **(B,D)**. Quantification was performed on three to four organoids per line with *n* = 31–159 measurements for patient *α* and *n* = 25–47 measurements for patient ω.

We first asked whether our genetically engineered cerebral organoids display an early AD phenotype to validate our model. Therefore, we conducted immunofluorescence analyses to confirm the genetic link between specific patterns of tau phosphorylation and *APOE3Ch* using anti-pTau S396 antibody. pTau S396 is an early marker of pathological tau phosphorylation (Mondragón-Rodríguez et al., 2014). pTau S396 staining was similar in Patient ω’s isogenic control organoids with the *PSEN1* E280A mutation and was reduced in *PSEN1*WT organoids (Figures 2A,B). When the *APOE3Ch* variant was introduced to Patient ω, pTau S396 staining was significantly reduced compared to isogenic patient controls and corrected tau phosphorylation to *PSEN1*WT levels (Figures 2A,B). This staining pattern was consistent in Patient α, whereby the removal of the *APOE3Ch* variant led to a significant increase in pTau S396 staining compared to all other Patient α cell lines (Figures 2C,D). We concluded that the *APOE3Ch* variant was able to produce a protective pattern of early tau phosphorylation defined as low pTau S396 in cerebral organoids derived from an AD-protected and non-protected patient within the *PSEN1* E280A background. Overall, the effects of *APOE* genotypes on tau phosphorylation were independent from *PSEN1* genotypes, suggesting a direct effect of *APOE* genotypes on the status of tau phosphorylation.

### Single-cell RNA sequencing of cerebral organoids reveals Wnt signaling differences

**Figure 3:**
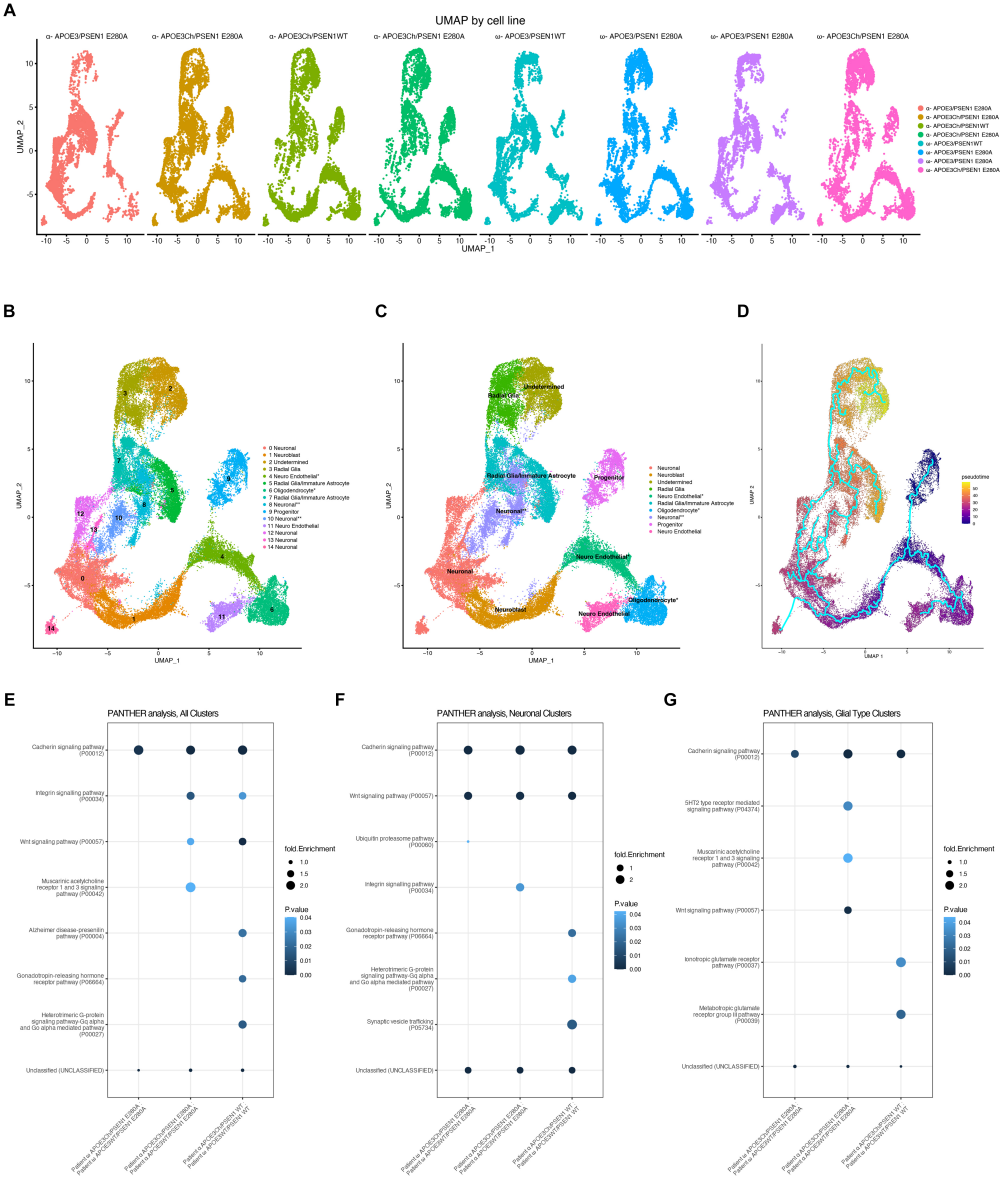
Cell line scRNA-seq UMAP cluster characteristics. scRNAseq data were downsampled and integrated using SCTransform and Harmony. Individual cell line UMAPs show an overlap, indicating that integration was successful **(A)**. Cell cluster transcript lists were run through GSEA C8 Cell Type for cluster identity **(B)**. ^*^ represents cell cluster identities defined by the second highest hit in GSEA C8 Cell Type analysis and the *post hoc* transcript expression profile, while ^**^ represents cell cluster identities defined by *post hoc* transcript expression profiles. Similar cluster identities were combined to form reference groups for downstream analysis **(C)**. Asterisk denotes cell cluster identity that was second hit in fGSEA due to its better representation of transcriptomic profile and differentiation protocol used **(B,C)**. Pseudotime was performed using the Monocle analysis suite to represent the differentiation timing to cell identity, initiating the analysis at Cluster 9 (Progenitor) as time zero **(D)**. PANTHER pathways analysis was performed on total cells, all clusters **(E)**, neuronal clusters 0, 1, 8, 10, 12, 13, and 14 **(F)**, and glia-type clusters 3, 5, and 7 **(G)**.

We conducted scRNA sequencing to identify potential dominant effects associated with *APOE* genotypes. The quality of datasets was confirmed within Seurat using standard QC measures (Supplementary Figure 3) and threshold set. To be able to compare the results within and between all cell lines, we downsampled all datasets to 6,400 cells after thresholding and QC and then integrated all scRNA-seq output using Harmony and SCTransform. UMAPs for both patients exhibit an overlap of cluster and cell types, indicating that integration was successful (Figure 3A). By using GSEA C8 Cell Type Signature Gene Sets, SCSA, and *post-hoc* transcript analysis, we were able to identify cell types (Figures 3B,C). Cell type identities in the SCSA analysis were mostly identical to the results obtained by GSEA C8 analysis; however, SCSA analysis revealed that clusters 5 and 7 are likely astrocyte or early astrocyte. We noticed a difference in cell clustering that any organoid containing *APOE3Ch* was more populated in the cluster 2 and 3 regions of the integrated uMAPs. We have identified that clusters 2 and 3 were also the last clusters in our developmental pattern and trajectory pseudotime analysis (Figure 3D), indicating that *APOE3Ch* likely influences cell developmental timings or cell type identity.

Next, we assessed the transcriptome of genes of interest to Alzheimer’s disease and resistance (Arboleda-Velasquez et al., 2019; Lopera et al., 2023), *APOE*, *APP*, *MAPT*, *PSEN1*, and *RELN* and found no notable differences across cell lines (Supplementary Figure 4). It should be noted that *APOE* displays minimal transcript expression in neuronal clusters and is enriched in glial Clusters 5 and 8 (Supplementary Figure 4), as expected.

Owing to the complexity and diverse genetic backgrounds of our cell lines, we first sought to identify differences across the datasets and to streamline workflow by converting datasets to pseudo-bulk to identify broad changes. Here, we identified that the Christchurch variant has a profound effect on Wnt signaling and Cadherin signaling pathways (Figure 3E), both of which utilize β-catenin. Next, we sought to assess pathway differences seen in the neuron clusters. When assessing neuronal-specific clusters, PANTHER pathways analysis yielded that the *APOE3Ch* variant influences Wnt signaling and Cadherin signaling, as well as others (Figure 3F). Due to the expression of *APOE* from glial cells, we queried these cell clusters revealing also Wnt signaling and Cadherin signaling, among others (Figure 3G). We then assessed the successful gene hits through the datasets and found that, for each patient, *APOE3Ch* drastically decreases transcript levels of *WNT2B*, *WNT4*, and *WNT7B* in our cerebral organoids but does not influence β-catenin transcript (Supplementary Figure 5). We also traced the *WNT2B*, *WNT4*, and *WNT7B* to maximum intensities in glial cell-type clusters (Supplementary Figure 5).

### β-Catenin is elevated in *APOE3Ch* cerebral organoids

**Figure 4:**
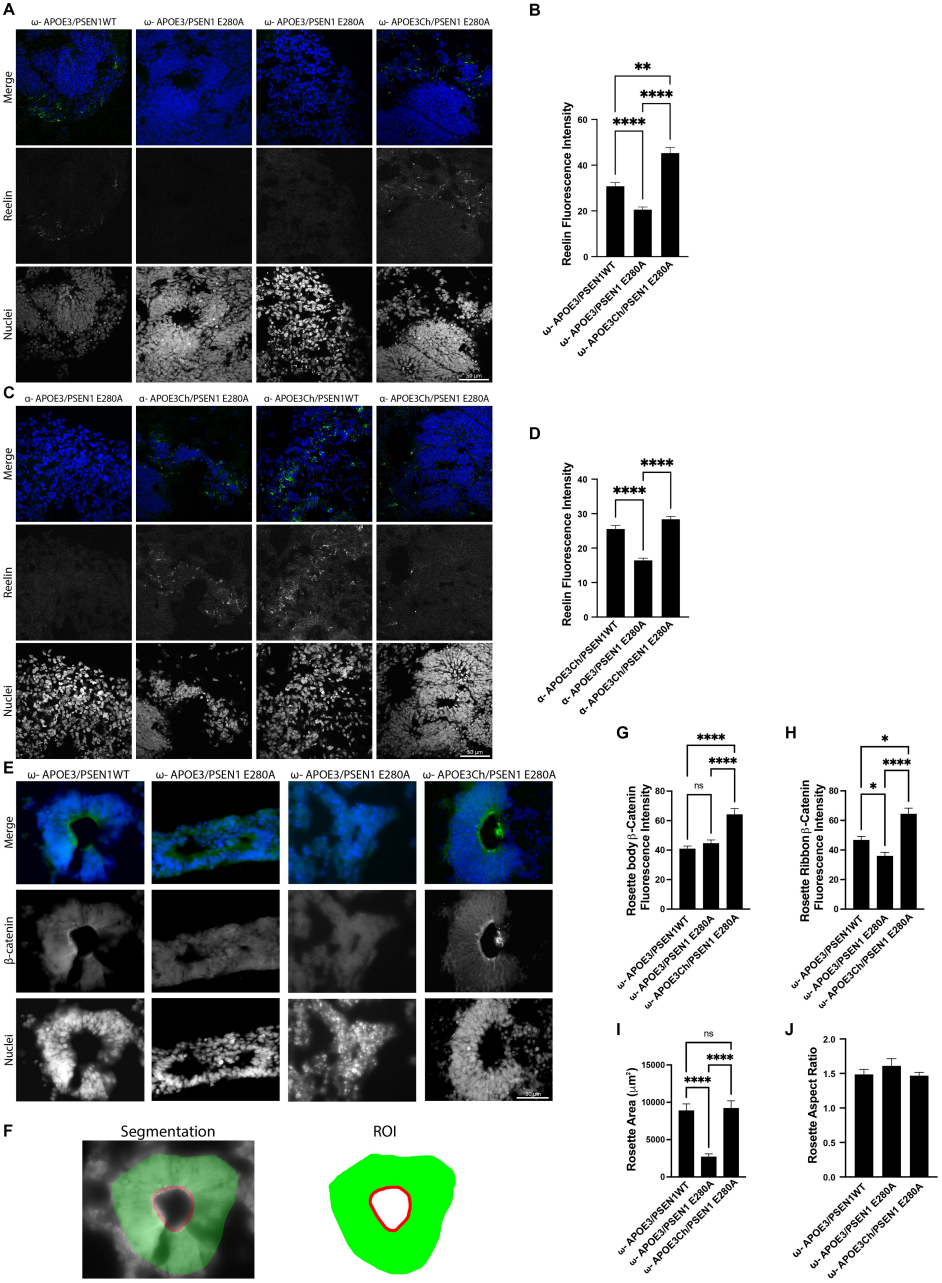
*APOE3Ch* influences reelin, β-catenin, and neural rosette features. Immunofluorescence staining was performed on cerebral organoids to identify changes in protein of interest and physical data. Organoids were imaged at 63X and quantified. Representative images of patient ω **(A)** and patient α **(C)** stained for reelin and nuclei. Reelin channel intensity was averaged, with isogenic controls averaged together for patient ω (*n* = 28–64 measurements per line) **(B)** and patient α (*n* = 44–120 measurements per line) **(D)** using three to four organoids per line. Cerebral organoids were stained for β-catenin using three to four organoids per line rosettes and were identified in patient ω (*n* = 17–29 measurements per line) and imaged at 63X **(E)**. Segmentation was performed, and regions of interest were defined **(F)**. β-catenin was quantified for both rosette body **(G)** and rosette ribbon **(H)**. Rosette physical features were then measured for area **(I)** and aspect ratio **(J)**. Scale bars for panels **A**, **C**, and **E**=50 μm.

Cerebral organoids have a high level of cellular heterogeneity, and we, therefore, focused on the characterization of rosettes, which are structures commonly observed in cerebral organoids resembling neural tubes that include pseudostratified epithelium with apico-basal polarity (Di Lullo and Kriegstein, 2017). Rosettes are an ideal morphogenetic model of brain development, with inner layers containing progenitors and more mature neurons toward the outer layers. Rosettes were smaller within *APOE3*/*PSEN1* E280A organoids compared to their *PSEN1*WT or *APOE3Ch* counterparts, suggesting a differential maturation phenotype. Consistent with a more mature phenotype, *APOE3Ch* organoids stained prominently with Reelin, a marker of more mature neurons (Figures 4A–D; Lancaster et al., 2013; Di Lullo and Kriegstein, 2017). Canonical Wnt activation leads to the accumulation of β-catenin and inhibition of GSK3β, a critical modulator of tau phosphorylation (Jackson et al., 2002; De Ferrari et al., 2014). Thus, we hypothesized that Wnt/β-catenin/Cadherin pathway regulation could link *APOE* genotypes to tau phosphorylation via modulation of β-catenin. Distribution of β-catenin was prominent in apical regions close to the rosette’s lumen (ribbon-like) and more homogenously localized within the pseudostratified epithelium of organoids with *APOE3Ch* variant (Figure 4E). We segmented the body of the rosette and ribbon for the quantification of β-catenin expression (Figures 4F–H). Rosette body and ribbon features show a significant increase of β-catenin expression in *APOE3Ch* variant carrier organoids vs. control. This phenotype was not impacted by *PSEN1* genotypes, suggesting a direct effect of the *APOE* genotype on tau phosphorylation phenotypes. We observed a marked decrease in the rosette area within *APOE3/PSEN1* E280A organoids compared to *APOE3Ch* rosettes (Figure 4I). This observation did not carry into the rosette aspect ratio (Figure 4J).

### Increased nuclear β-catenin in neurons of protected brain regions of patient α

**Figure 5:**
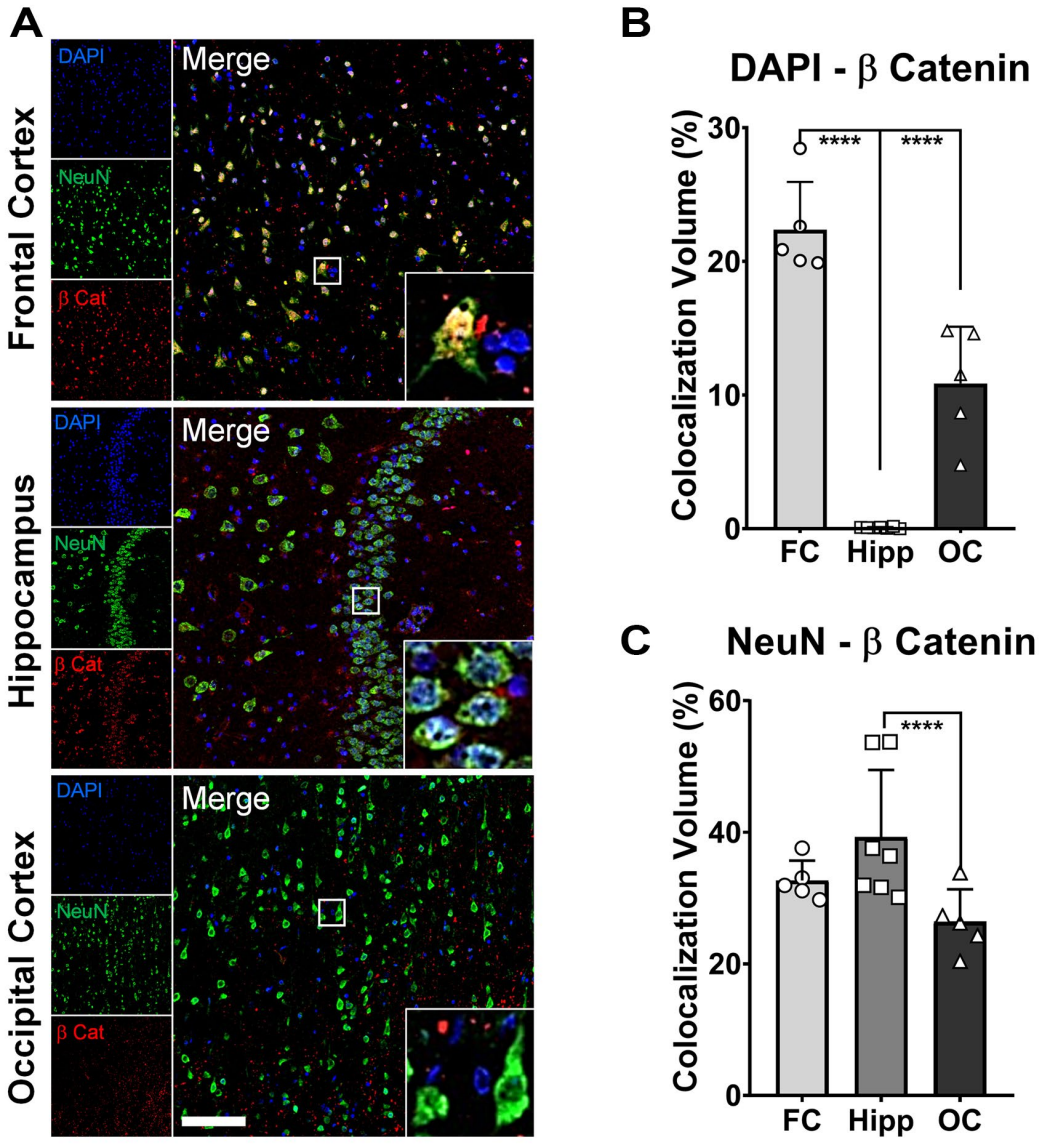
Increased nuclear β-catenin in neurons of protected brain regions of patient α. Representative immunofluorescence (IF) micrographs of the frontal cortex (FC), hippocampus (Hipp), and occipital cortex (OC) stained for β-catenin (red), NeuN (green), and cell nuclei (DAPI, blue). Insets present magnified images of neurons showing the degree of colocalization between the three markers. Scale bar = 100 μm. **(A)**. Bar graphs for colocalization analysis depicting thresholded colocalization volumes (TCVs) between DAPI and β-catenin in FC, Hipp, and OC. The percentage of β-catenin colocalizing in nuclei is significantly higher in FC than in both structures, Hipp and OC (one-way ANOVA, *p* < 0.0001 for both) **(B)**. Bar graphs for colocalization analysis depicting TCVs between DAPI and β-catenin in FC, Hipp, and OC. The percentage of β-catenin colocalizing with neurons is significantly higher only in Hipp when compared to OC (one-way ANOVA, *p* = 0.025) **(C)**.

Previously, we have reported a comprehensive *postmortem* analysis of brain tissue collected from a homozygous *APOE3Ch PSEN1* E280A carrier. Three brain areas were selected as representative for the degree of protection conferred by this *APOE* mutation: frontal cortex (FC), hippocampus (Hipp), and occipital cortex (OC) (Sepulveda-Falla et al., 2022). As a validation of our current results in brain organoids derived from the same patient, we performed colocalization analysis between β-catenin, DAPI as a nuclear marker, and NeuN as a neuronal marker. We found that the FC showed a significantly higher thresholded volume of colocalization (TVC) for β-catenin in nuclei when compared to Hip and OC (Figures 5A,B), indicating a higher level of activation of the β-catenin pathway in this brain region. On the other hand, TVC for β-catenin in neuronal cells only showed statistically significant differences between Hip and OC (Figures 5A,C), possibly reflecting neuronal loss and general neurodegeneration described in this brain area from this patient. These results indicate a potential link between Wnt signaling, β-catenin, and AD protection.

### ApoE3Ch acts as a Wnt3a signaling enhancer

Together, our findings revealed an intriguing correlation: a reduced expression of multiple Wnt ligands alongside a significant increase in β-catenin protein expression. This correlation prompted us to investigate the presence of a hypothetical activator of the pathway in our system. If a putative activator was present, the Wnt ligand downregulation may arise as a compensatory mechanism.

**Figure 6:**
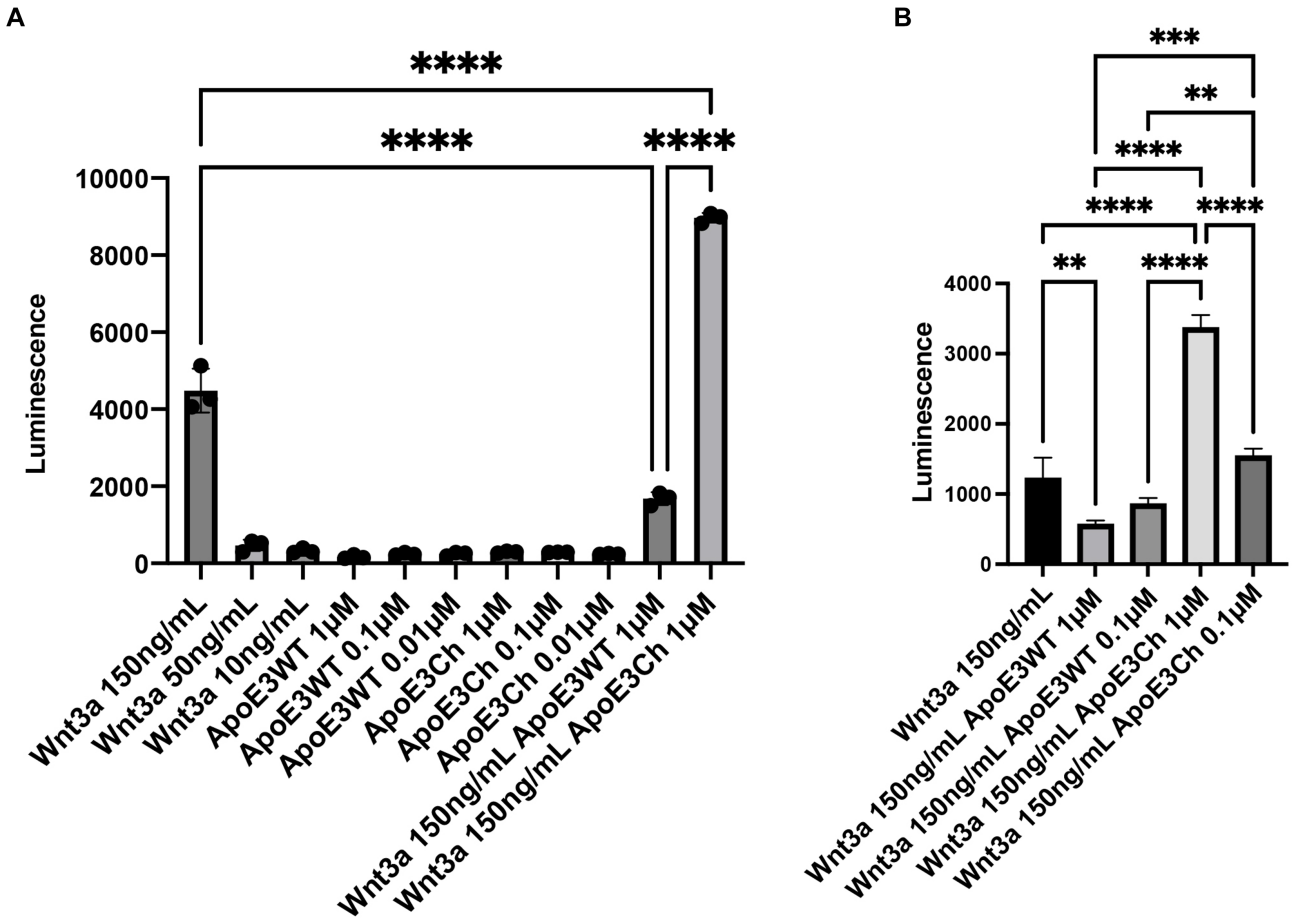
ApoE3Ch acts as a Wnt3a signaling enhancer. HEK293 cells with TCF/LEF luciferase reporter element cells were tested against ApoE3 WT, ApoE3Ch, and Wnt3a to assess Wnt signaling activation in triplicate. Cells were treated individually with each compound and in combination **(A)**. Activation was confirmed in a secondary experiment **(B)**. Statistical significance using a one-way ANOVA and post-hoc Tukey’s test to consider a *p*-value of < 0.05 significance.

Therefore, we examined whether the ApoE3Ch protein could directly influence Wnt signaling. To explore this influence, we utilized a Wnt reporter cell line, validated for Wnt3a. Our initial investigations indicated that ApoE3WT and ApoE3Ch alone did not induce Wnt signaling. However, when combined with Wnt3a ligands, ApoE3Ch acted as a Wnt signaling activator, while ApoE3WT functioned as a Wnt signaling inhibitor (Figure 6A). This finding was further confirmed through a repeated experiment, which also revealed a dose-dependent relationship (Figure 6B). Notably, while the inhibitory effect of ApoE on Wnt signaling, attributed to competitive binding to LRP receptors, has been reported before with ApoE4 being the stronger inhibitor compared to ApoE3 and ApoE2 (Caruso et al., 2006), the discovery of ApoE3Ch as a Wnt3a activator represents a new and unexpected gain-of-function property of this rare variant (neomorphism).

We tested other Wnt ligands including Wnt5a and Wnt16b, but they could not be accurately measured in our assay (data not shown). Taken together, these findings suggest that ApoE3Ch might operate through multiple mechanisms upstream of tau phosphorylation, including the regulation of Wnt signaling.

## Discussion

We have developed novel iPS cell lines derived from the ADAD Paisa kindred, used genetic engineering to correct the *PSEN1* E280A mutation as well as editing native *APOE3* to either add or remove the Christchurch variant, formed cerebral organoids, identified pathways through scRNA-seq, and supported these findings through immunostaining. While it is well documented that iPS cell systems can model aspects of AD pathology (Penney et al., 2020; Nelson et al., 2023), we have also demonstrated here that the iPS system is also capable of accurately identifying cellular processes differentially regulated by the *APOE3Ch* variant. We have found that the *APOE3Ch* variant alters the translational landscape to promote changes in Cadherin and Wnt signaling, which affects β-catenin, irrespective of the *PSEN1* background. We have also found that ApoE3Ch is an enhancer of Wnt signaling. Taken together, this system has accurately identified cellular processes historically implicated in AD pathogenesis that are affected by the *APOE3Ch* variant. Additional studies will be required to provide therapeutic intent to AD as well as other neurodegenerative diseases and tauopathies.

Our findings in the *postmortem* tissue of Patient α, a homozygous *APOE3Ch PSEN1* E280A carrier, confirm the relevance of the Wnt/β-catenin pathway as a putative mechanism of protection. A pathological downregulation of this pathway has been described in *postmortem* studies in the frontal cortex of Alzheimer’s patients (Folke et al., 2019). Interestingly, the frontal cortex, the most protected brain region with the highest *APOE* expression levels in the Patient α *APOE3Ch PSEN1* E280A carrier showed higher activation of the Wnt/β-catenin pathway. This finding bolsters our previous suggestion for an *APOE* dose-dependent protective effect (Sepulveda-Falla et al., 2022).

We were puzzled by the finding of Wnt ligand downregulation in the context of increased β-catenin signaling. To further explore this apparent discrepancy, we hypothesized that the changes arose from the presence of a hypothetical Wnt activator in our system that led to the compensatory decrease of endogenous Wnt ligands. As a first suspect, we tested whether ApoE3Ch could itself act as a Wnt activator using a reporter assay. This turned out to be the case as indicated by our data. ApoE3Ch protection may operate via multiple mechanisms, one of which is a neomorphism.

Our findings revealed a novel and unexpected gain-of-function property of ApoE3Ch, showing its ability to enhance Wnt3a signaling activity. While ApoE3Ch alone did not induce Wnt signaling, intriguingly, when combined with the Wnt3a ligand, it acted as a Wnt3a signaling activator. In contrast, ApoE3 WT displayed a Wnt signaling inhibitory effect under the same conditions. Our study sheds light on the intricate regulatory roles of ApoE isoforms in modulating the Wnt signaling pathway, providing insights into potential mechanisms underlying the resistance to tauopathy observed in Patient α, the ApoE3Ch-carrying individual with the *PSEN1* E280A mutation. These findings may have significant implications for the development of therapeutic strategies targeting Wnt signaling in Alzheimer’s disease and other tauopathies. Further investigations are warranted to fully elucidate the molecular mechanisms underlying ApoE3Ch-mediated Wnt activation and its relevance to neuroprotection in the context of tau-related pathologies.

*PSEN1* mutations can affect neuronal differentiation in iPS cell-derived systems, such as cerebral organoids, through reduced Notch signaling and premature aging phenotypes (Arber et al., 2021). Our study found developmental differences in organoids carrying the *PSEN1* E280A genotype, including abnormal tau phosphorylation at a young developmental stage. This suggests that young cerebral organoids can be used as pathological models for AD and as a tool to study the mechanisms of protection.

The effects of *APOE3Ch* on Wnt and Cadherin signaling uncovered by scRNA sequencing of cerebral organoids were unexpected and may operate via multiple mechanisms, ultimately resulting in β-catenin upregulation. Cadherins are a family of calcium-dependent transmembrane adhesion proteins that link β- and α-catenin to the actin cytoskeletal network (Punovuori et al., 2021) and also regulate cellular homeostasis through signaling mediating development, proliferation, apoptosis, and disease pathology (Yulis et al., 2018). Cadherins regulate calcium-dependent cell–cell adherent junctions, where the chelation of calcium abolishes adhesive activity and allows proteolytic degradation of cadherins (Nagar and Overduint, 1996; Kim et al., 2011). Thus, proper calcium levels play a vital role in cell–cell dynamics as well as maintaining a pool of cadherin.

Wnt signaling influences multiple cellular processes such as cell fate determination, cell polarity, organogenesis, stem cell renewal (Komiya and Habas, 2008), neuronal health (Inestrosa and Varela-Nallar, 2014), autophagy (Pérez-Plasencia et al., 2020), and phagocytosis and ferroptosis (Wang et al., 2022). Wnt signaling has also been implicated in neurological aging (Inestrosa et al., 2020) and neurological aging disorders such as AD (Palomer et al., 2019). ApoE has also been implicated in Wnt signaling regulation (Zhao et al., 2023). ApoE is known to be produced in both radial glia and other glia cell populations (Zhao et al., 2023); however, neurons are known to produce ApoE under stress (Konings et al., 2021). Our scRNA-seq analysis revealed that our cerebral organoids produced ApoE but displayed minimal expression in neuronal clusters and selective enrichment in glial population clusters.

Wnt signaling can be assigned into two pathways, canonical Wnt signaling or the non-canonical planar cell polarity (PCP) and Wnt/calcium pathway subdivisions. Canonical Wnt signaling requires extracellular Wnt binding to LRP5/6 and Frizzled for signal transduction across the cell membrane to Disheveled. Once internalized, the signal is passed to the β-catenin destruction complex, a proteinaceous structure composed of GSK3β and other proteins, resulting in the release of β-catenin by the inhibition of GSK3β. Free β-catenin is then able to translocate to the nucleus and activate TCF/LEF transcription. In the PCP pathway, Wnt directly binds to Frizzled and transduces the signal to Disheveled, which in turn activates RhoA and Rac1 and eventual JNK pathways. In the non-canonical calcium-dependent subpathway, Wnt binds directly to Frizzled, transduces the signal to Disheveled, and interacts with trimeric G proteins and phospholipase C, increasing intracellular calcium concentration inducing CamKII and calcineurin activation (Inestrosa and Varela-Nallar, 2014). CaMKII is vital in controlling NMDA receptor activity (Incontro et al., 2018), which also acts as a calcium channel (Lau et al., 2009).

GSK3β is a protein kinase that phosphorylates and primes tau for inclusion in paired helical filaments and fibrils (Hooper et al., 2008). Indeed, GSK3β is known to phosphorylate tau at the early pathology site S396 (Li and Paudel, 2006), and GSK3β is also a vital component of the β-catenin destruction complex. It is responsible for phosphorylating β-catenin for ubiquitination and proteosomal degradation (Inestrosa and Varela-Nallar, 2014). This persistent degradation maintains low levels of free cytoplasmic β-catenin and inhibits gene transcription. Cerebral GSK3β stimulation by phosphorylation at Y216 is mediated by intracellular calcium levels and calcium-dependent PYK2 (Hartigan and Johnson, 1999; Hartigan et al., 2001; Sayas et al., 2006).

*APOE* is the most significant known risk factor for sporadic Alzheimer’s disease, ApoE4 exhibits the strongest receptor binding and is considered a high-risk allele, while ApoE2 exhibits the weakest receptor binding and is considered protective (Yamazaki et al., 2019). The ApoE3Ch variant was found in a protected ADAD subject and was shown to have weaker binding than its ApoE3 WT counterpart to heparin sulfate proteoglycans (Arboleda-Velasquez et al., 2019). However, imputation of genetic causality was also not feasible because of the rarity of the Christchurch variant. Thus, the need for genetic analyses *ex vivo* was conducted here.

In this study, we have demonstrated that *APOE3Ch* produces a reduction of pTau S396 phosphorylation in an AD cerebral organoid model and that ApoE3Ch enhances Wnt signaling. However, it is important to note that further studies with more mature organoids will be necessary to confirm the protective tau phosphorylation pattern persists over time to determine efficacy within the *PSEN1* E280A background. Wnt signaling is also modulated by additional factors such as Wnt ligand, Fzd receptors, sFRP, R-Spondin, and Dkk, where our validation study relied on the HEK293 system only; therefore, further studies will be required to understand the secretome of *APOE3Ch* cerebral organoids and functional mechanisms of signaling enhancement (e.g., HSPG binding; Marino et al., 2023). CRISPR was employed to generate the genetic edits desired for this study, and while optimal guidance motifs were selected to reduce off-target edits, we cannot rule out any potential off-target effects, though isogenic controls were used to minimize this potential limitation. Developmental patterns of tau phosphorylation may also present a confounding factor. Due to the dynamic nature of a developing system and that gene expression patterns are on a continuum, cluster cell identification has its limitations.

In sum, our data suggest that iPS-derived cerebral organoids can be informative in the identification of biological processes influenced by protective mutations. Our model shows a link between *APOE3Ch* and a protective pattern of early pathogenic tau phosphorylation. Importantly, our data showed a prominent role for Wnt and Cadherin signaling in the presence of the *APOE3Ch* variant. β-catenin is differentially regulated in *APOE3Ch* cerebral organoids, which is known to affect Wnt/Cadherin signaling and GSK3β activity, and confirmed by *postmortem* analysis of the ApoE3Ch patient. Furthermore, we confirmed scRNA-seq findings through ApoE3Ch enhancement of Wnt signaling. These findings are relevant in informing the sphere of influence associated with AD protection mediated by the *APOE3Ch* variant and serve to build the toolbox for identifying therapeutic targets against AD.

## Data availability statement

The names of the repository/repositories and accession number(s) can be found in the article/Supplementary material. Sequencing data used in this manuscript are available through Gene Expression Omnibus (GEO) (Accession GSE241453) at https://www.ncbi.nlm.nih.gov/geo/query/acc.cgi?acc=GSE241453.

## Ethics statement

The studies involving humans were approved by University of Antioquia IRB and Mass General Hospital IRB. The studies were conducted in accordance with the local legislation and institutional requirements. The participants provided their written informed consent to participate in this study.

## Author contributions

PP: Data curation, Formal analysis, Investigation, Methodology. TV: Formal analysis, Investigation. GV: Formal analysis, Investigation.JS: Formal analysis, Investigation. NV-M: Formal analysis, Investigation. CM: Formal analysis, Investigation. SK: Investigation, Formal analysis. MMP: Resources. DA: Resources. MJ-D-R: Resources. AB: Resources. DS-F: Formal analysis, Investigation. FL: Project administration, Resources, Supervision. YTQ: Project administration, Investigation, Supervision. JFA-V: Conceptualization, Investigation, Methodology, Project administration, Supervision. RM: Conceptualization, Formal analysis, Investigation, Methodology, Project administration.

## Footnotes

^1^ https://www.gsea-msigdb.org/gsea/msigdb/index.jsp

^2^ https://cole-trapnell-lab.github.io/monocle3/docs/trajectories
