# Supplementary figures and images for: APOE3 Christchurch modulates β-catenin/Wnt signaling in iPS cell-derived cerebral organoids from Alzheimer’s cases
Source: Front Mol Neurosci. 2024 Mar 20;17:1373568. doi: 10.3389/fnmol.2024.1373568 (PMC10987717; doi:10.3389/fnmol.2024.1373568)

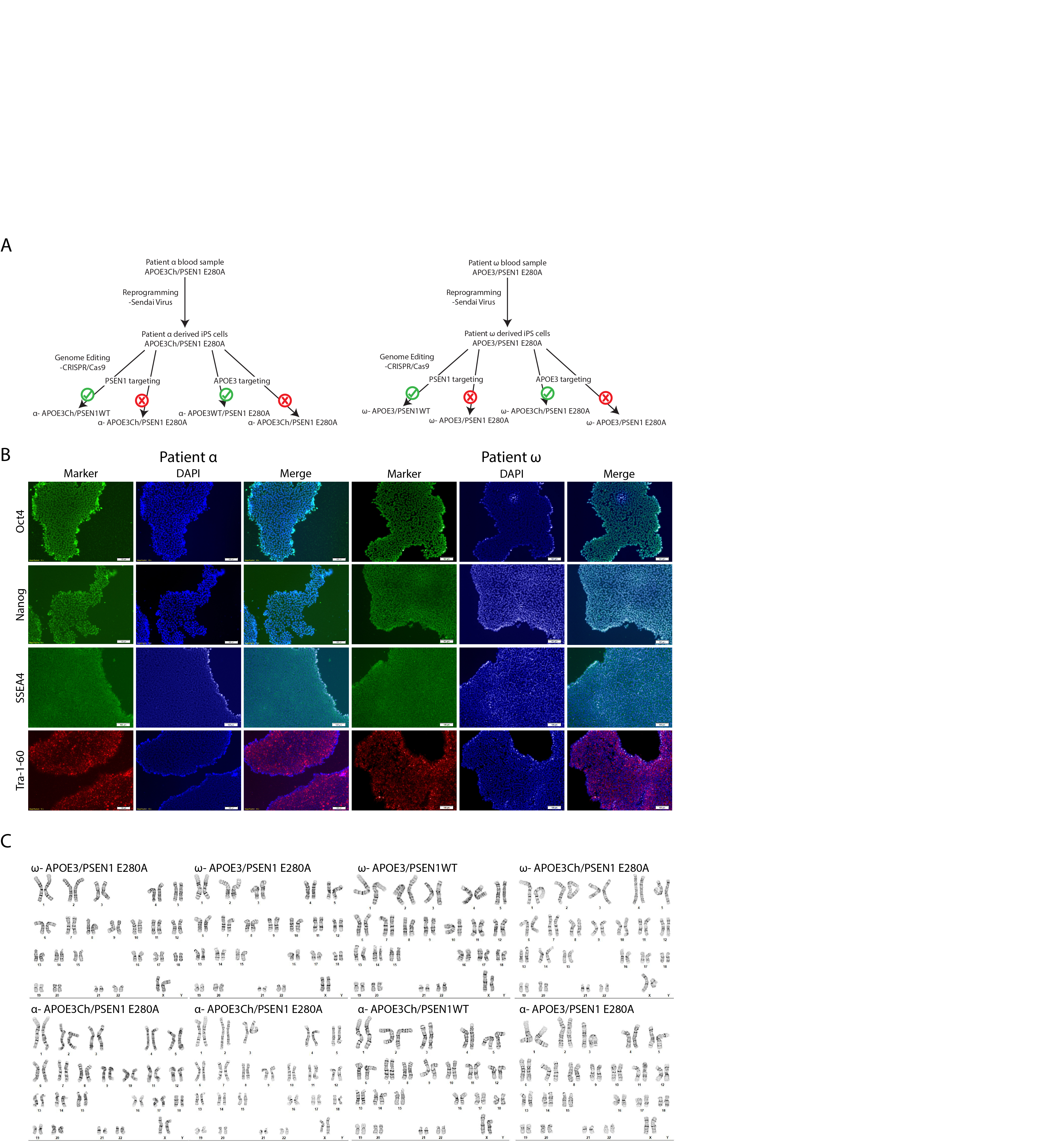

Supplement: Supplementary file 1 [file Image_1.JPEG]

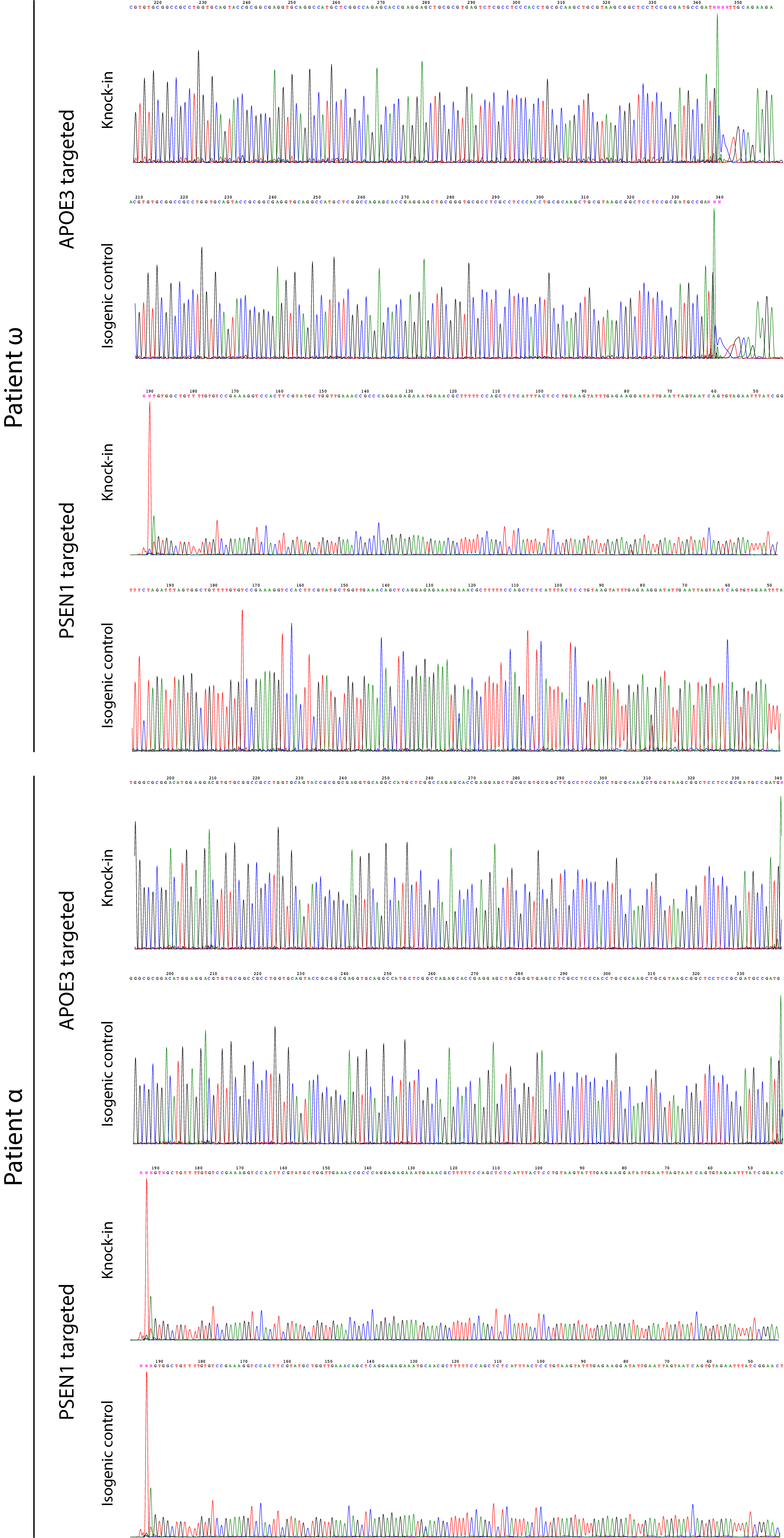

Supplement: Supplementary file 2 [file Image_2.JPEG]

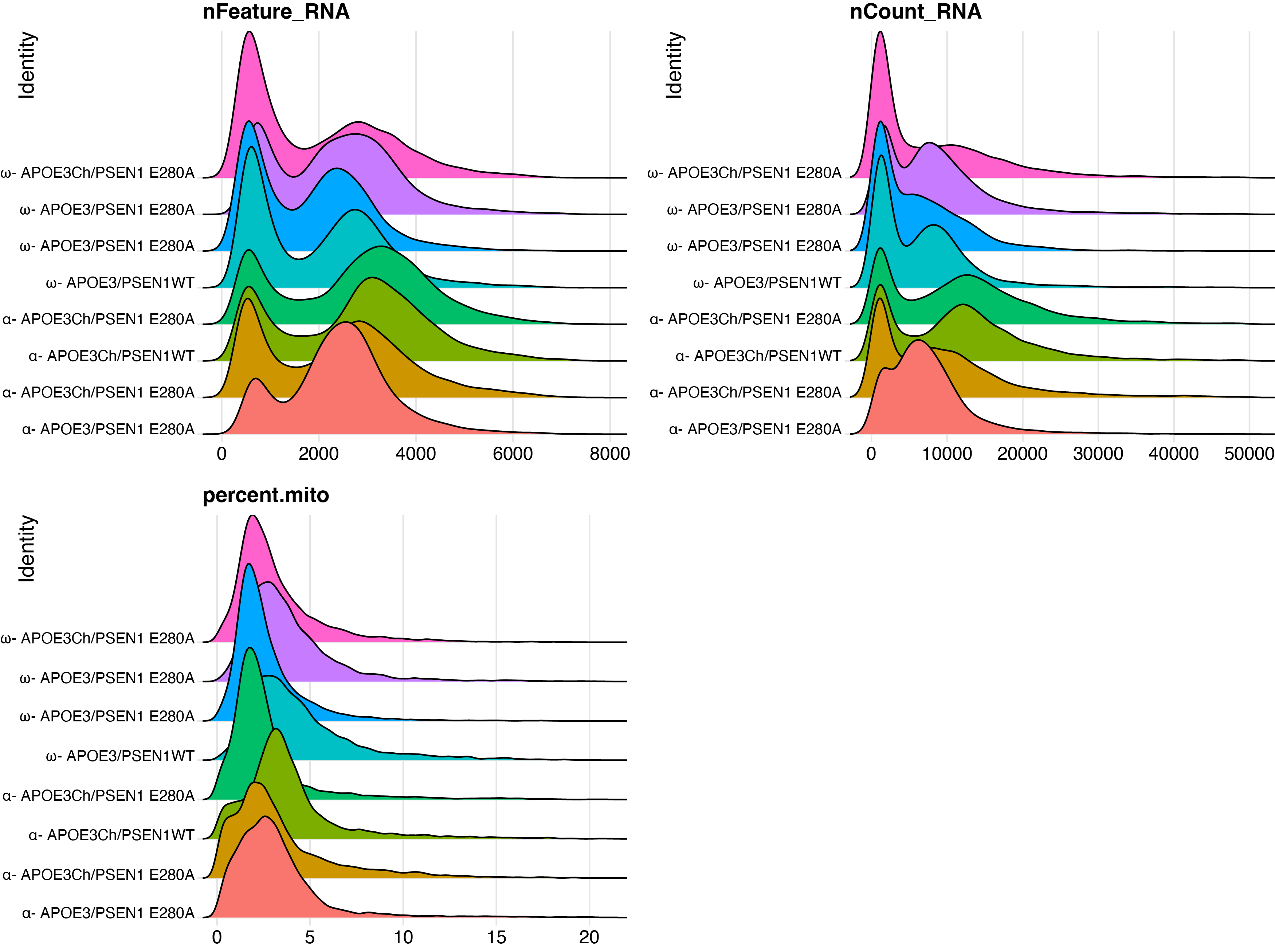

Supplement: Supplementary file 3 [file Image_3.JPEG]

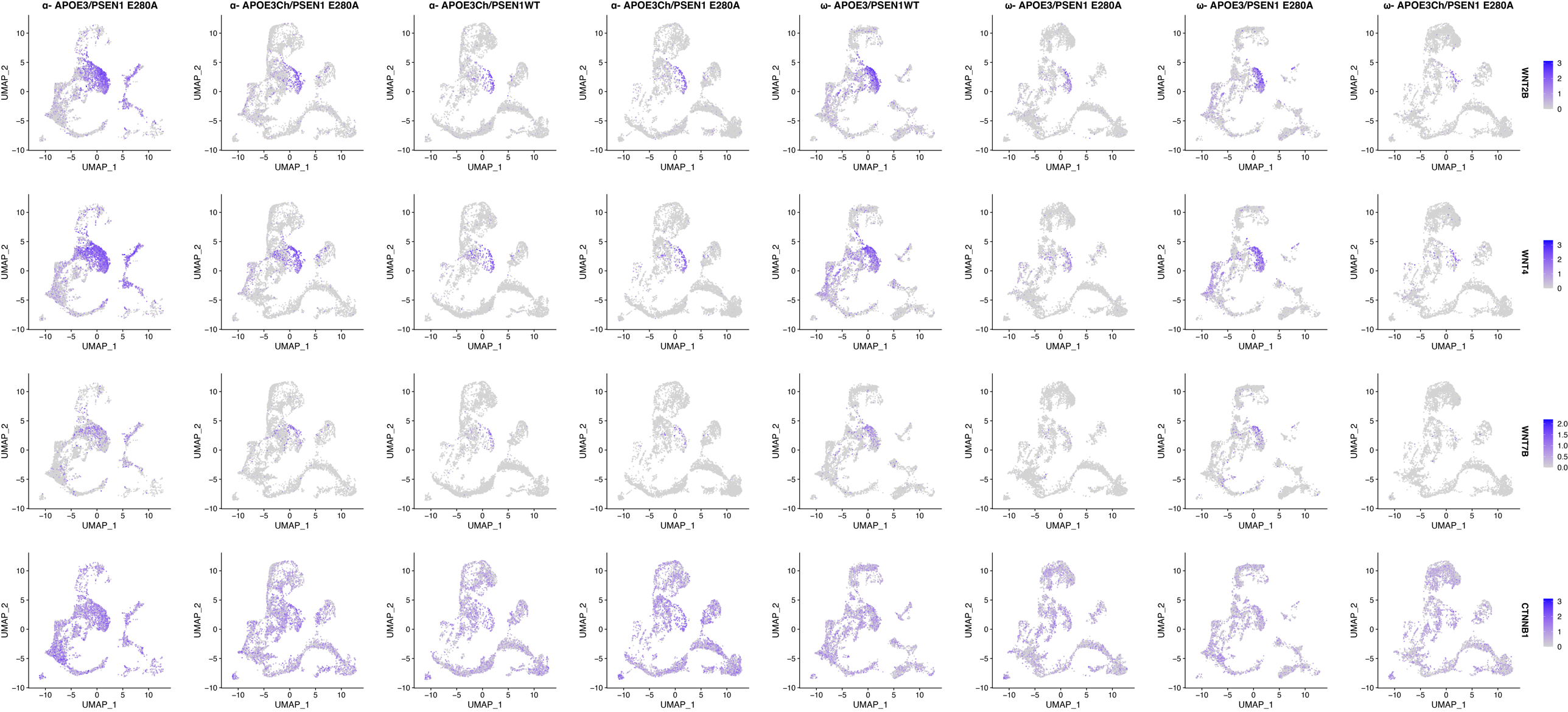

Supplement: Supplementary file 5 [file Image_5.JPEG]
